# Epigenetic synergy between decitabine and platinum derivatives

**DOI:** 10.1186/s13148-015-0131-z

**Published:** 2015-09-11

**Authors:** Taichun Qin, Jiali Si, Noël J-M Raynal, Xiaodan Wang, Vazganush Gharibyan, Saira Ahmed, Xin Hu, Chunlei Jin, Yue Lu, Jingmin Shu, Marcos RH Estecio, Jaroslav Jelinek, Jean-Pierre J. Issa

**Affiliations:** Department of Leukemia, University of Texas MD Anderson Cancer Center, Houston, TX 77030 USA; Department of Epigenetics and Molecular Carcinogenesis, University of Texas MD Anderson Cancer Center, Houston, TX 77030 USA; Harbin Institute of Hematology & Oncology, Harbin, 150010 China; Fels Institute for Cancer Research and Molecular Biology, Temple University, 3307 North Broad Street, Rm 154, PAHB, Philadelphia, PA 19140 USA

**Keywords:** 5-aza-2′-deoxycytidine, Carboplatin, DNA methylation, Epigenetic reactivation, Tumor suppressor genes

## Abstract

**Background:**

Aberrant epigenetic silencing of tumor suppressor genes has been recognized as a driving force in cancer. Epigenetic drugs such as the DNA methylation inhibitor decitabine reactivate genes and are effective in myeloid leukemia, but resistance often develops and efficacy in solid tumors is limited. To improve their clinical efficacy, we searched among approved anti-cancer drugs for an epigenetic synergistic combination with decitabine.

**Results:**

We used the YB5 cell line, a clonal derivative of the SW48 colon cancer cell line that contains a single copy of a hypermethylated cytomegalovirus (CMV) promoter driving green fluorescent protein (GFP) to screen for drug-induced gene reactivation and synergy with decitabine. None of the 16 anti-cancer drugs tested had effects on their own. However, in combination with decitabine, platinum compounds showed striking synergy in activating GFP. This was dose dependent, observed both in concurrent and sequential combinations, and also seen with other alkylating agents. Clinically achievable concentrations of carboplatin at (25 μM) and decitabine reactivated GFP in 28 % of the YB5 cells as compared to 15 % with decitabine alone. Epigenetic synergy was also seen at endogenously hypermethylated tumor suppressor genes such as *MLH1* and *PDLIM4*. Genome-wide studies showed that reactivation of hypermethylated genes by the combination was significantly better than that induced by decitabine alone or carboplatin alone. Platinum compounds did not enhance decitabine-induced hypomethylation. Rather, we found significantly inhibited HP1α expression by carboplatin and the combination. This was accompanied by increased histone H3 lysine 4 (H3K4) trimethylation and histone H3 lysine 9 (H3K9) acetylation at reactivated genes (*P* < 0.0001) and reduced occupancy by methyl-binding proteins including MeCP2 and methyl-CpG-binding domain protein 2 (MBD2) (*P* < 0.0001).

**Conclusions:**

Our results suggest that the combination of decitabine with platinum analogs shows epigenetic synergy that might be exploited in the treatment of different cancers.

**Electronic supplementary material:**

The online version of this article (doi:10.1186/s13148-015-0131-z) contains supplementary material, which is available to authorized users.

## Background

Epigenetic changes have been recognized in the past decade as major drivers of different types of cancer [1, 2]. The involvement of DNA methylation and histone modifications in aberrant gene silencing has particular functional roles in malignant progression. Aberrant DNA methylation is frequently observed in human cancer and contributes to malignant transformation by silencing multiple tumor suppressor genes [2]. DNA methylation at gene promoters recruits methyl-binding proteins (MBPs) that are associated with histone deacetylases (HDACs) as well as histone methyltransferases [3, 4]. Methylation at the histone H3 lysine 9 (H3K9) residue is considered as a key element of promoting epigenetic silencing by recruiting heterochromatin protein 1 (HP1) in a repressive complex that modulates chromatin structure locally as well as excludes transcription factors [5, 6]. On the other hand, trimethylation at histone H3 lysine 4 (H3K4) and acetylation at H3K9 residues are associated with gene transcription [7].

Understanding of epigenetic alterations in cancer led to treatment by targeting epigenetic modulators, an approach referred to as epigenetic therapy [1]. Several epigenetic drugs including DNA hypomethylation inducers and histone deacetylase inhibitors are now approved by the Food and Drug Administration for cancer treatment. Also, combinations of these two types of drugs that achieved more epigenetic reactivation are being tested in several clinical trials [8–10]. 5-Aza-2′-deoxycytidine (decitabine), a DNA hypomethylating drug, is approved for the treatment of the myelodysplastic syndrome (MDS) [11]. Decitabine is a deoxycytidine analog. After incorporating into DNA, it traps DNA methyltransferase in the form of a covalent protein-DNA adduct, leading to DNA replication-dependent hypomethylation [11].

Despite the rapid development of epigenetic therapy, problems remain including primary and secondary resistance to epigenetic drugs and rare responses in solid tumors [12–14]. Therefore, it is necessary to identify more efficient approaches in epigenetic drug development as well as to develop better combination therapy. Based on the hypothesis that activation of silenced gene expression is part of the mechanism of action of these drugs [15–17], we established the YB5 cell line derived from the SW48 colon cancer cell line to develop a screening system for epigenetic drug reactivation in cancer [18, 19]. YB5 contains a hypermethylated cytomegalovirus (CMV) promoter driving a green fluorescent protein (GFP) reporter. YB5 has a stably integrated single copy of this transgene, and the locus is transcriptionally silent, with high levels of DNA methylation, histone deacetylation, and nucleosome occupancy [18, 19]. Hypomethylating drugs reactivate the CMV promoter and drive GFP expression to high levels which can be easily scored by flow cytometry analysis. Here, we used this system to test whether known anti-cancer drugs have epigenetic effects alone or could enhance decitabine epigenetic therapy. We discovered an epigenetic synergy between platinum analogs and decitabine, which we trace to HP1α degradation and chromatin remodeling.

## Results

### Decitabine and platinum analogs synergistically activate GFP

**Fig. 1:**
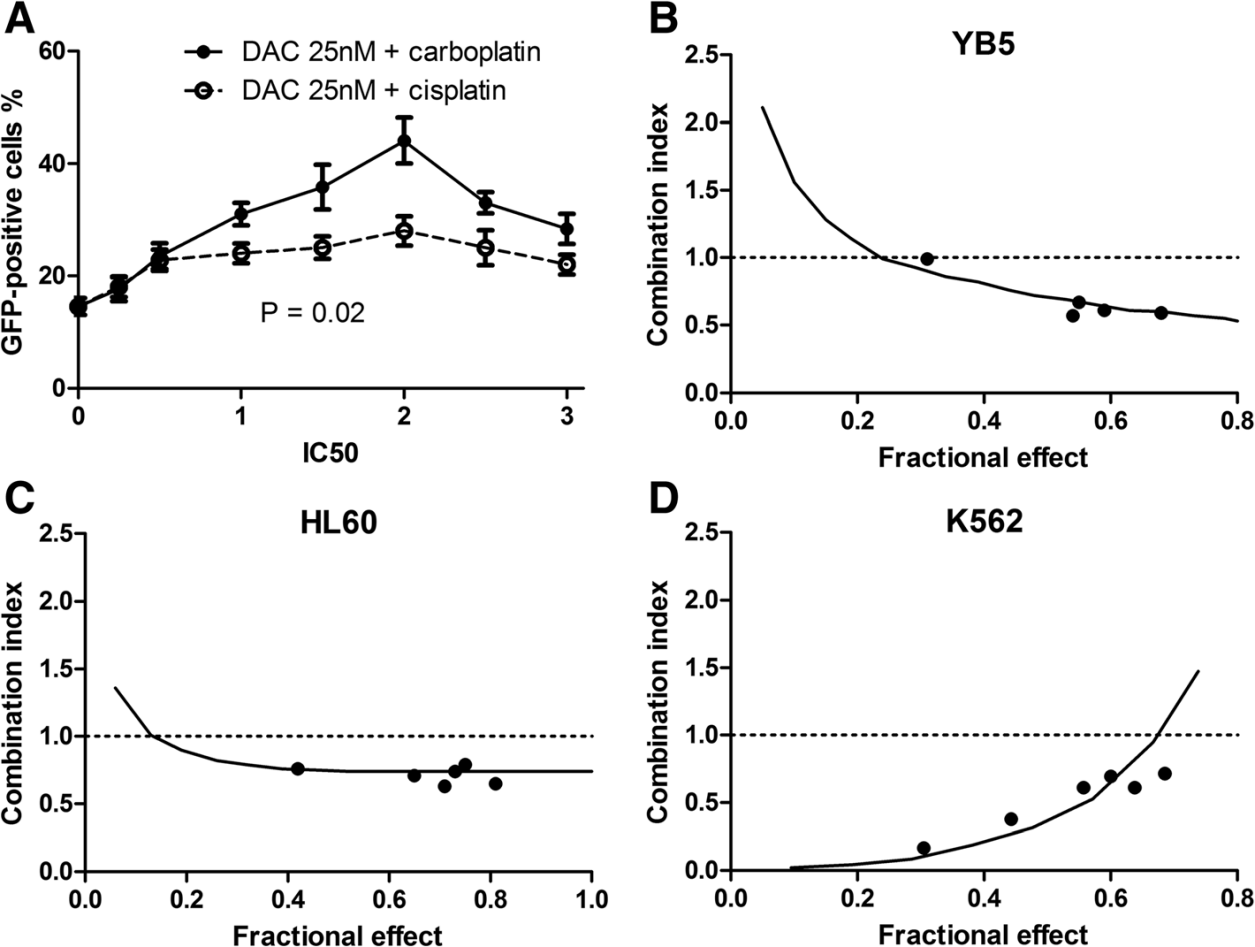
Gene expression and cell killing synergy of decitabine in combination with carboplatin. **a**. Effects of the combination of decitabine with platinum analogs on GFP reactivation. YB5 cells were treated daily for 4 days with a fixed dose of decitabine at 25 nM (1/2 IC50) plus 0.5 to 3 IC50 doses of carboplatin or cisplatin, respectively. GFP was measured after the end of treatment by flow cytometry analysis. Standard deviation was calculated based on two independent experiments. **b**–**d** Combination index (*CI*) plots of decitabine and carboplatin combinations in human cancer cell lines. The cells were treated with decitabine alone, carboplatin alone, and decitabine plus carboplatin in fixed molar ratios based on the IC50 values of each drug and incubated for 4 days. We used the ratios of 1/8, 2/8, 3/8, 4/8, 6/8, and 8/8 IC50 for decitabine and carboplatin for each respective cell line. We assessed cell viability by counting of cells excluding trypan blue. The effects of combinations were estimated using the CalcuSyn software, based on the median-effect method. CI < 1 indicates synergy, CI ~ 1 indicates additive effect, and CI > 1 means antagonism

We initially selected 18 commonly used anti-cancer drugs to test for GFP reactivation including anthracycline antibiotics, alkylating agents, topoisomerase inhibitors, antimicrotubule agents, antitumor antibiotics, and antimetabolites (Table 1). None of the 16 evaluable drugs significantly activated GFP by themselves (the two tested anthracyclines were autofluorescent and thus could not be evaluated for GFP reactivation). By contrast, decitabine showed GFP activation ranging from 15 % at 25 nM up to 40 % at higher doses, as previously reported [18]. We next examined the combination of decitabine at a fixed low dose of 25 nM (equal to 1/2 IC50) with a variety of doses of the anti-cancer drugs. One category of drugs that strikingly enhanced GFP reactivation by decitabine was platinum analogs (Table 1). The IC50 of carboplatin and cisplatin in YB5 cells was 25 and 2 μM, respectively. The combination of decitabine at 25 nM with carboplatin or cisplatin at doses of 0.5, 1.0, 1.5, 2, 2.5, and 3 times the IC50 value showed a synergistic effect on GFP reactivation. GFP % gradually increased and peaked at 2× IC50 of carboplatin and then decreased at higher doses (Fig. 1a). At the optimal dose, carboplatin tripled the amount of GFP+ cells. At the clinically achievable dose of 25 μM, carboplatin increased GFP expression from 15 to 28 %. Carboplatin was more efficient at boosting GFP reactivation than cisplatin, possibly because carboplatin has a bidentate dicarboxylate ligand as its leaving group instead of the more labile chloride ligands, and hence, its effects are longer lasting [20].

**Table 1:** A list of anti-cancer drugs used for screening of GFP reactivation

Drugs (dose range tested)	Category	Conc. producing the highest GFP	GFP % ± SEM drug alone	GFP ratio (±SEM)^a^
Carmustine (50–100 nM)	Alkylating agent	50 nM	0.1 ± 0.1	0.86 ± 0.15
Mechlorethamine (50–100 nM)	Alkylating agent	100 nM	0.2 ± 0.2	1.82 ± 0.1^c^
Carboplatin (50–75 μM)	Alkylating agent	75 μM	0.4 ± 0.1	1.86 ± 0.83^c^
Oxaliplatin (5–20 μM)	Alkylating agent	20 μM	2.2 ± 2.0	1.58 ± 1.1
Cisplatin (200–500 nM)	Alkylating agent	500 nM	0.1 ± 0.1	1.07 ± 0.14
Chlorambucil (10–50 μM)	Alkylating agent	50 μM	0.3	1.1
Temozolomide (50–75 μM)	Alkylating agent	75 μM	0.2	1.28^b^
Fludarabine (10 μM)	Antimetabolite	10 μM	0.1 ± 0.1	0.99 ± 0.45
Cytarabine	Antimetabolite	10 nM	0.1	1.21 ± 0.24
Gemcitabine (100–400 nM)	Antimetabolite	200 nM	0.9	0.86
Fluorouracil (100–400 nM)	Antimetabolite	200 nM	0.2 ± 0.1	0.48 ± 0.28
Clofarabine (100–500 nM)	Antimetabolite	100 nM	0.1	0.04
Irinotecan (25–100 nM)	Topoisomerase I inhibitor	25 nM	0.2 ± 0.1	1.47 ± 0.12^c^
Etoposide	Topoisomerase I inhibitor	100 nM	0.2	0.87 ± 0.07
Paclitaxel (1–10 nM)	Antimicrotubule agent	10 nM	2.2 ± 0.8	0.92 ± 0.04
Mitomycin C	Antitumor antibiotic	100 μg/mL	1.1 ± 0.8	1.1 ± 0.1
Doxorubicin	Anthracycline antibiotic		ND (autofluorescent)	
Idarubicin	Anthracycline antibiotic		ND (autofluorescent)	

In these experiments, anti-cancer drug treatment (for 24 h) was performed following decitabine pre-treatment at 100 nM for 72 h. GFP ratio was calculated for each experiment by dividing the GFP signal obtained for the drug combination by the signal produced by decitabine alone for each experiment

^a^Data shown were selected for doses producing the highest GFP percentage (±SEM)

^b^Higher concentrations of temozolomide of 200 and 250 μM in combination with decitabine produced respectively 30 and 41.4 % of GFP positive cells

^c^A significant increase in GFP ratio

We also performed sequential treatment of decitabine followed by anti-cancer drugs. YB5 cells were treated with several doses of each anti-cancer drug either alone for 24 h or following a 72-h pre-treatment with decitabine at 100 nM. After decitabine pre-treatment alone, 13 ± 0.9 % of YB5 cells expressed GFP. Several of the drugs enhanced GFP expression including carboplatin and alkylating agents such as mechlorethamine and temozolomide (at high doses) that also significantly increased GFP activation by decitabine, suggesting a class effect. Interestingly, irinotecan, a topoisomerase I inhibitor, also modestly increased GFP reactivation by decitabine (Table 1).

We next examined the effects of combining decitabine and carboplatin on cell viability in YB5 and in two leukemia cell lines (HL60 and K562). We used fixed ratios of 1/8, 2/8, 3/8, 4/8, 6/8, and 8/8 IC50 for decitabine and carboplatin in all respective cell lines. The combination of decitabine and carboplatin was synergistic in all tested cell lines, indicated by combination index (CI) values <1 (Fig. 1b–d). For example, the CI value of the combination treatment ranged from 0.58 to 0.98 in YB5 cells, from 0.67 to 0.82 in HL60, and from 0.2 to 1.0 in K562 cell line. No antagonism (CI > 1.0) was observed.

### Decitabine and carboplatin synergistically activate transcription of silenced genes

**Fig. 2:**
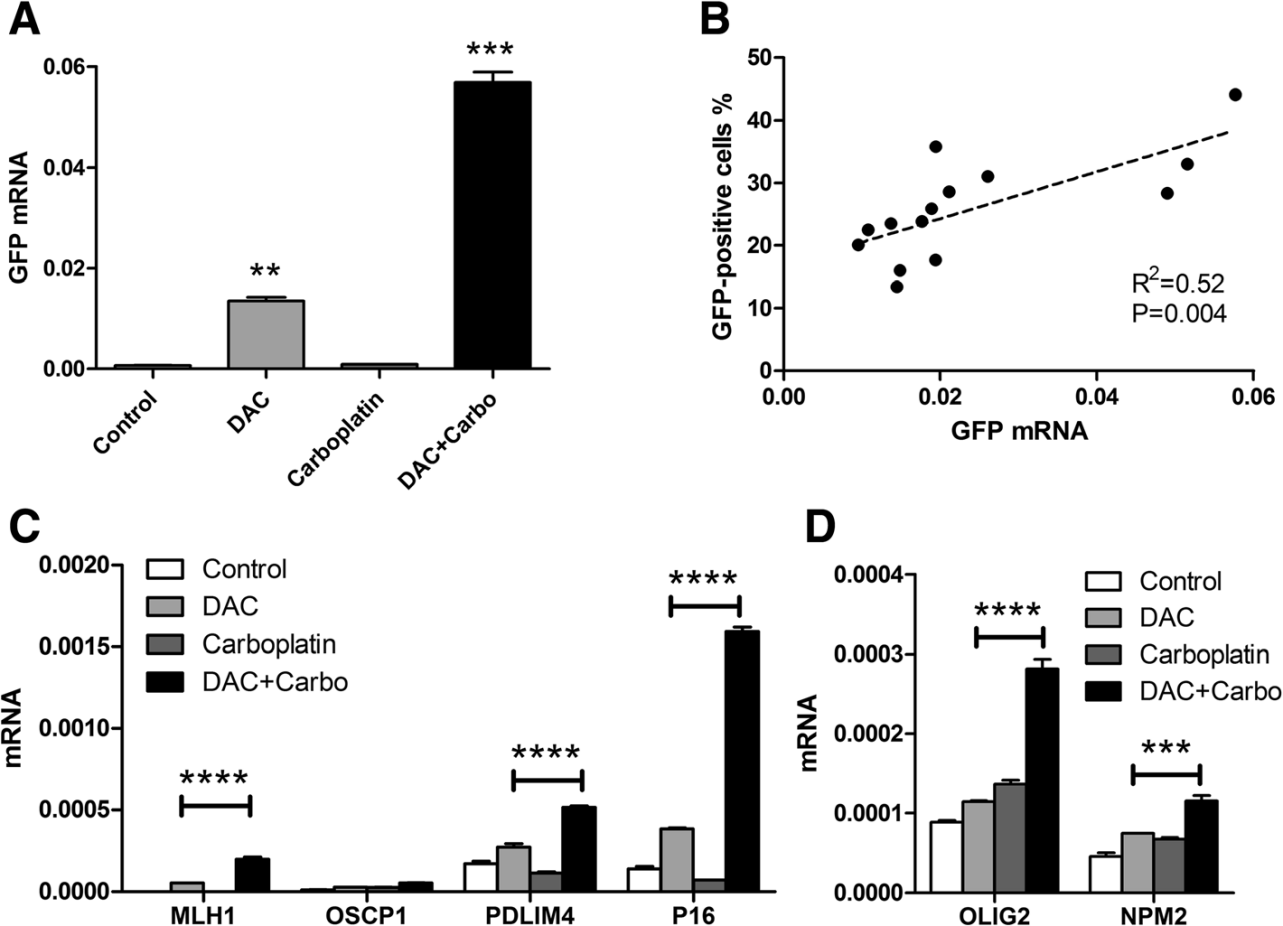
Carboplatin enhanced gene transcription activated by decitabine. **a**. Carboplatin enhanced GFP mRNA expression in decitabine-treated cells. YB5 cells were treated with decitabine 25 nM, carboplatin 25 μM, and decitabine + carboplatin for 4 days. GFP mRNA was measured by qPCR and normalized to GAPDH. **b**. The correlation of GFP % measured by flow cytometry with GFP mRNA expression. We treated YB5 cells with a fixed dose of 25 nM decitabine and a variety of doses of carboplatin or cisplatin. The *broken line* shows fit to a linear regression model. **c**. Reactivation of expression of genes with methylated promoters in YB5. YB5 cells were treated with decitabine 25 nM, carboplatin 25 μM, and decitabine + carboplatin for 4 days. **d**. Reactivation of expression of genes with methylated promoters in HL60. HL60 cells were treated with decitabine 200 nM, carboplatin 25 μM, and decitabine + carboplatin for 4 days. mRNA expression was measured by qPCR and normalized to GAPDH. Statistical significance of Bonferroni-corrected *t* tests is shown by *asterisks* (**P  < 0.01, ***P < 0.001, ****P < 0.0001)

In this cellular model, effects of decitabine on gene expression are through transcription [18]. To determine whether this was also the case for the apparent synergy, we used quantitative reverse transcription polymerase chain reaction (qRT-PCR) to examine mRNA expression of GFP and of endogenously silenced genes. Compared to control cells, decitabine at a low dose (25 nM) activated expression of GFP mRNA 10-fold, carboplatin at 25 μM showed no significant activation, while the combination of decitabine with carboplatin induced expression 32-fold (*P* < 0.001, Fig. 2a). Furthermore, the number of GFP fluorescent cells determined by flow cytometry correlated well with GFP mRNA expression in YB5 cells treated with different doses of carboplatin or cisplatin in combination with decitabine (*R*^2^ = 0.52, *P* = 0.004, Fig. 2b). Thus, the synergy appeared to be transcriptional, suggesting epigenetic effects. We next determined whether GFP mRNA expression could be a valid surrogate for reactivation of endogenously silenced genes in YB5 cells, and whether this was relevant in another cell type (HL60, a leukemia cell line). We selected for analysis genes that showed promoter DNA hypermethylation and transcriptional repression. Using qRT-PCR, we found that the addition of carboplatin to decitabine enhanced activation of *MLH1*, *PDLIM4*, and *p16* (*CDKN2A*) expression in YB5 (Fig. 2c) as well as of *OLIG2* and *NPM2* in HL60 (Fig. 2d).

**Fig. 3:**
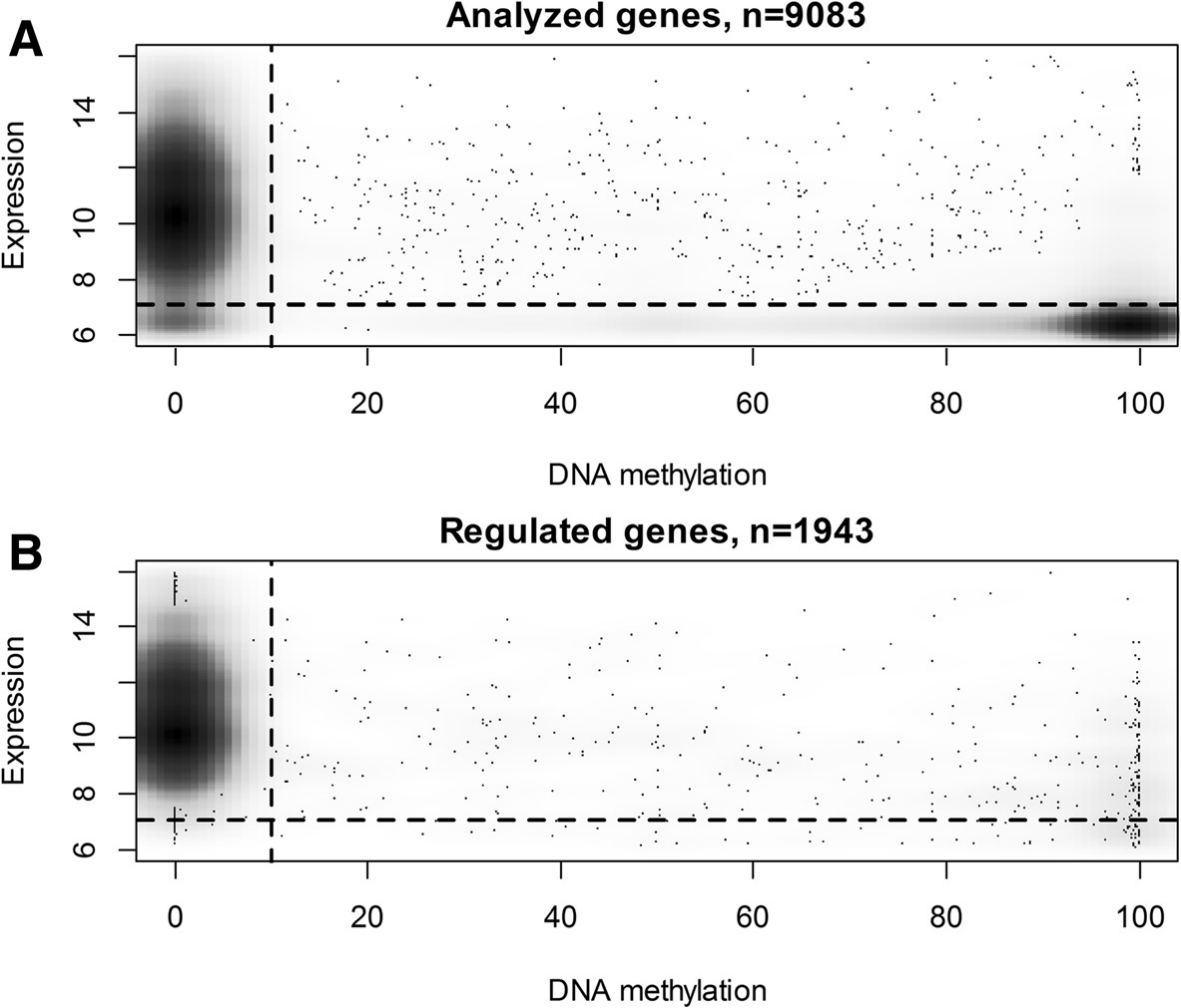
Baseline gene expression and DNA methylation in YB5 cells. **a**. Percent DNA methylation at CpG sites within ±500 bp from TSS of 9083 genes (*x*-axis) vs gene expression at baseline (*y*-axis). **b**. Baseline methylation vs expression for the subset of regulated genes showing expression changes ≥1.5-fold after treatment with decitabine, carboplatin, or their combination. The *broken line* divides the data into four quadrants: (*Q1*) *upper left*, unmethylated expressed genes; (*Q2*) *upper right*, methylated expressed genes; (*Q3*) *lower left*, unmethylated silenced genes; and (*Q4*) *lower right*, methylated silenced genes

**Fig. 4:**
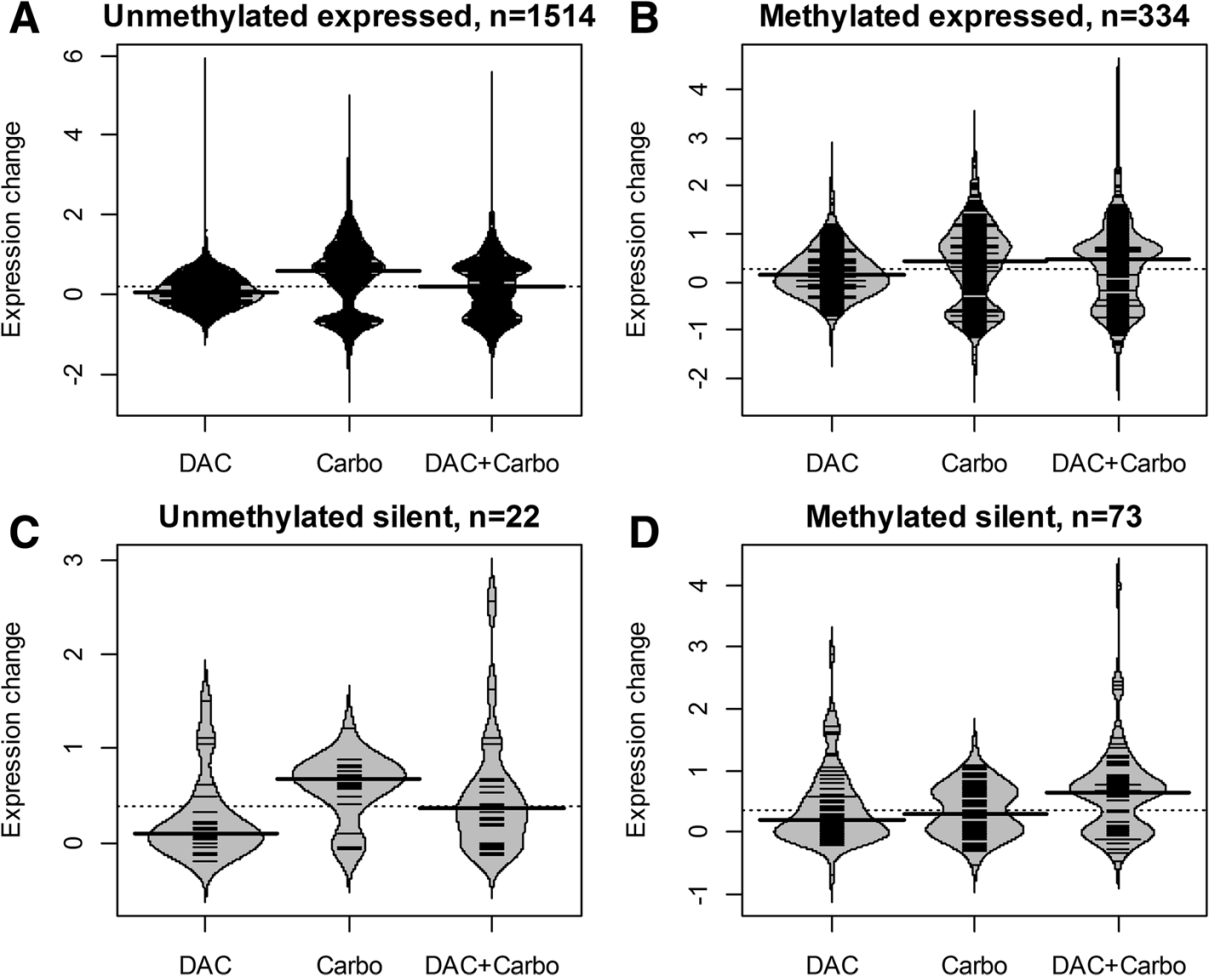
Gene expression changes in YB5 cells after drug treatment. *Beanplots* show changes of expression in 1943 regulated genes compared to baseline after treatment of YB5 cells with decitabine 25 nM (DAC), carboplatin 20 μM (Carbo), and their combination (DAC + Carbo) in the four quadrants defined in Fig. 3. The *width of the shape sacks* represents histograms of data density. The *short lines* inside the shapes depict individual data points. *Thick horizontal bars* show medians. The *dotted line* behind the shapes shows median of data in all three columns. The expression changes are plotted in log2 units. **a**–**c** Carboplatin treatment results in the largest gene expression changes. **d** Reactivation of methylated silenced genes by combined treatment with decitabine and carboplatin

We next analyzed the effect of decitabine and carboplatin on reactivation of hypermethylated genes at a genomic scale. We combined data on gene expression microarrays of untreated and drug-treated YB5 cells with genome-wide DNA methylation data generated using the quantitative, reduced representation-based method called digital restriction enzyme analysis of methylation (DREAM) [21], which queried the methylation status of 9083 gene promoters. Drug treatment consisted of decitabine alone at 25 nM, carboplatin alone at 25 μM, or the two drugs given concurrently. At baseline, there was a strong inverse correlation (*P* < 2 × 10^−16^) between promoter methylation and gene expression as expected, thus confirming the accuracy of the genome-wide measurements. A plot of expression vs methylation showed data distribution in four quadrants: (Q1) unmethylated and expressed (5697 genes), (Q2) methylated and expressed (1119 genes), (Q3) unmethylated and silenced (479 genes), and (Q4) methylated and silenced (1788 genes) (Fig. 3a, Table 2). Because both decitabine and carboplatin are expected to have effects on gene expression independent of DNA methylation, we analyzed the data in each quadrant separately. We found 1943 genes showing at least 1.5-fold expression change compared to the baseline (Fig. 3b) and analyzed the gene expression changes in each quadrant (Fig. 4). Carboplatin treatment had the greatest effect on the expressed genes (top three quartiles of gene expression). It increased expression of 13.3 and 13.4 % of unmethylated (quadrant 1) and methylated genes (quadrant 2), respectively. Functional annotation analysis revealed enrichment for mitochondrial genes and apoptosis (Additional file 1: Table S1). Carboplatin treatment also decreased expression of 6.4 and 7.2 % of unmethylated and methylated genes in quadrants 1 and 2, respectively (Table 2). Downregulated genes were significantly enriched for association with cell division, cell cycle, and DNA replication (Additional file 1: Table S2). Combined treatment with decitabine and carboplatin showed significantly enhanced expression of methylated silenced genes (quadrant 4). Decitabine alone (at this very low dose) upregulated expression of 1.2 % genes, carboplatin upregulated 1.6 % genes, while decitabine plus carboplatin combined upregulated 2.5 % (44 of 1778) methylated silenced genes (Table 2). Functional annotation analysis of the genes upregulated by the combination showed enrichment for positive regulation of apoptosis, cell adhesion, and integrin signaling (Additional file 1: Table S3).

**Table 2:** Effect of drug treatment on gene expression in YB5 cells

Methylation	Expression	Quadrant	Analyzed genes	Regulated genes	Change	DAC	Carbo	DAC + Carbo	*P* value^*^
<10 %	≥7.08	Q1	5697	1514	Up	177	755	527	<0.0001
					Down	58	365	312	<0.0001
≥10 %	≥7.08	Q2	1119	334	Up	64	150	149	<0.0001
					Down	14	81	61	<0.0001
<10 %	<7.08	Q3	479	22	Up	4	15	7	0.0224
					Down	0	0	0	n/a
≥10 %	<7.08	Q4	1788	73	Up	21	29	44	0.0119
					Down	1	0	0	n/a

^*^Chi-square test

Thus, at this very low dose, decitabine had a modest effect on the expression of unmethylated genes while activating a subset of repressed genes. Carboplatin (used at the IC50 dose) both activated and repressed unmethylated genes, and showed significant synergy with decitabine for those genes showing a high degree of promoter methylation (i.e., epigenetic synergy).

### Epigenetic synergy is independent of DNA demethylation

**Fig. 5:**
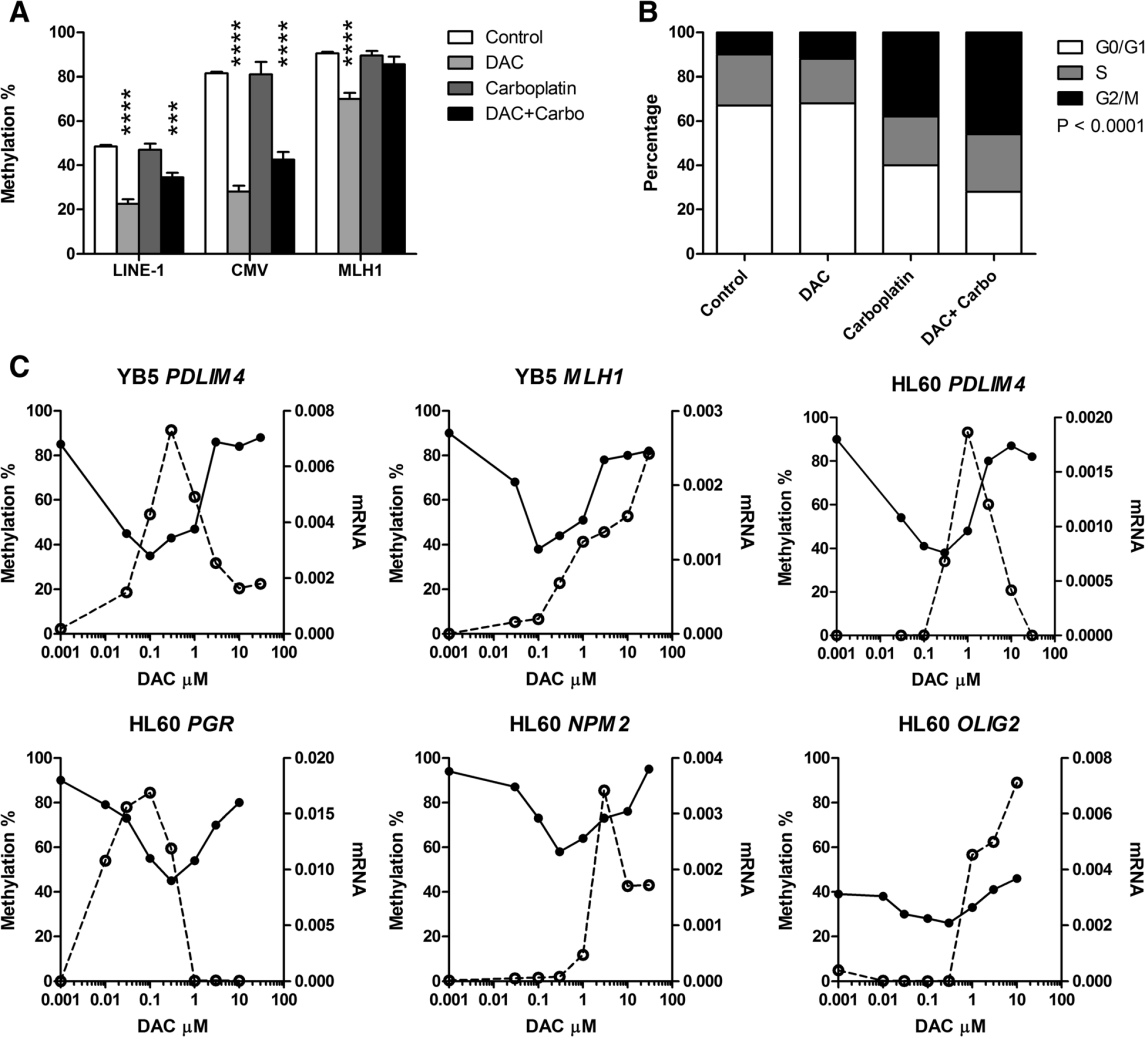
Correlation between gene expression and hypomethylation. **a**. Carboplatin did not induce hypomethylation of DNA. YB5 cells were treated with decitabine 25 nM, carboplatin 25 μM, and decitabine + carboplatin for 4 days, respectively. DNA methylation at LINE-1, CMV, and *MLH1* promoter CpG islands was analyzed by bisulfite pyrosequencing. Statistical significance of Bonferroni-corrected *t* tests is shown by *asterisks* (***P < 0.001, ****P < 0.0001). **b**. Carboplatin induced cell cycle arrest in the G2/M phase. YB5 cells were treated as described above and cell cycle distribution in G0/G1, S, and G2/M was measured by flow cytometry after propidium iodide staining and analyzed with the Chi-square test (*P* < 0.0001). **c**. Decitabine induced hypomethylation and gene expression in a dose-dependent manner. YB5 and HL60 cells were treated with a variety of logarithmically equally spaced concentrations of decitabine ranging from 0.03 to 30 μM. DNA methylation (*left x-axis*, *solid lines*) and mRNA expression (*right x-axis*, *dotted lines*) of *MLH1*, *PDLIM4*, *PGR*, *OLIG2*, and *NPM2* genes were measured by bisulfite pyrosequencing and qPCR, respectively

To search for mechanisms of enhanced gene transcription by the combination, we first examined DNA methylation. Carboplatin at 25 μM alone had no effects on methylation of the long interspersed nuclear element 1 (LINE-1) repetitive elements across the genome or on the CMV promoter methylation. After treatment with decitabine at 25 nM, LINE-1 methylation decreased from 48 to 22 %. When carboplatin at 25 μM was added to decitabine, it decreased only to 35 % compared with decitabine alone (Fig. 5a). Similarly, CMV methylation was 82 % in untreated YB5 cells as measured by bisulfite pyrosequencing. Decitabine treatment decreased methylation to 28 %, but the addition of carboplatin to decitabine dampened the decrease to 42 % (Fig. 5a). A possible explanation for the reduced hypomethylating effect is that carboplatin induced cell cycle arrest, resulting in less incorporation of decitabine into DNA and less inhibition of DNA methyltransferase activity. Indeed, flow cytometry analysis showed that carboplatin induced cell cycle arrest in the G2/M phase in a dose-dependent manner (Fig. 5b). The G2/M proportion in control and decitabine-treated cells was 10 and 12 %, respectively, but the addition of carboplatin at 25 μM increased it to 38 and 46 %, respectively. By contrast, the percentages of G0/G1 cells in control and decitabine-treated cells were 67 and 68 %, respectively, but the addition of carboplatin decreased this to 40 and 28 %, respectively (*P* < 0.0001, chi-square test).

These data show that synergy in gene activation cannot be explained by enhanced hypomethylation of promoter DNA. Generally, decitabine induces hypomethylation at low doses and cytotoxic effects or a DNA damage response that limits hypomethylation at higher doses [12]. It remains unclear how the hypomethylation response at different doses correlates with gene activation, and the carboplatin synergy data suggests a potential disconnect between the two. To test this further, we examined DNA methylation and reactivated gene expression at different doses. As previously reported [22], decitabine-induced hypomethylation followed a U-shaped pattern in YB5 and HL60 (Fig. 5c), with inhibited hypomethylation induction at higher drug concentrations. The doses of decitabine that induced maximum hypomethylation were the same for all the genes tested in the respective cell line (Fig. 5c). For example, decitabine at 100 nM induced maximum hypomethylation for *PDLIM4* and *MLH1* in YB5 while 300 nM induced maximum hypomethylation of *PDLIM4*, *PGR*, *NPM2*, and *OLIG2* in HL60. However, the gene expression response to decitabine was quite variable. Some genes (*PDLIM4* and *PGR*) followed a strict inverse correlation between methylation and expression, but for others (*MLH1* and *OLIG2*), we observed a different pattern whereby high drug concentrations induced a high degree of activation, despite dampening of the hypomethylation response (Fig. 5c), just as observed when carboplatin was added to decitabine.

### Decitabine and carboplatin synergy in chromatin remodeling

**Fig. 6:**
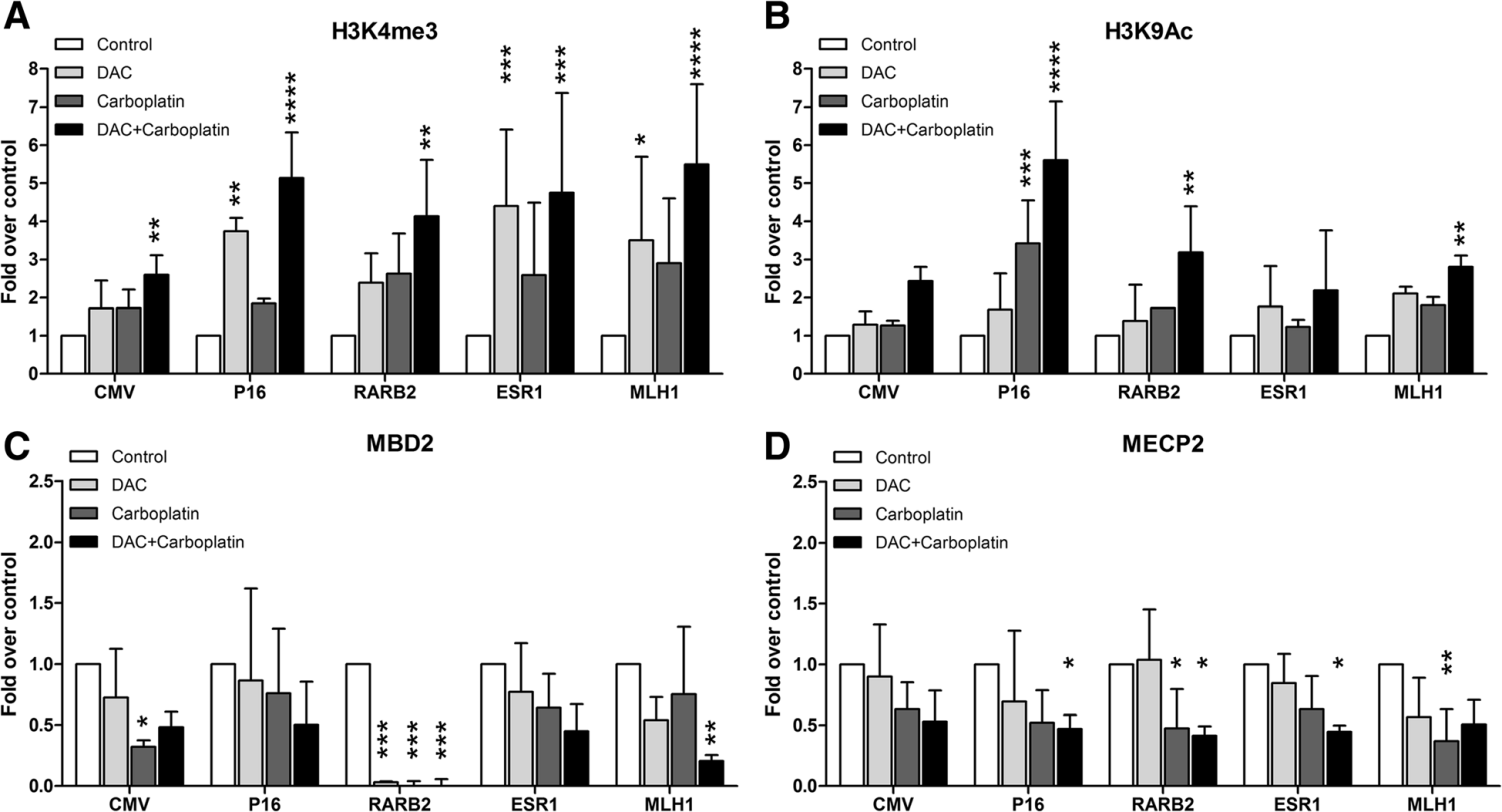
Decitabine and carboplatin modulate histones and methyl-binding proteins. We treated YB5 with 25 nM decitabine and/or 25 μM carboplatin for 4 days. Subsequently, we performed chromatin immunoprecipitation in YB5 controls and treated cells with antibodies against H3K4me3 (**a**), H3K9Ac (**b**), MBD2 (**c**), and MECP2A (**d**). We analyzed the abundance of the activation histone marks (H3K4me3, H3K9Ac) and methyl-binding proteins (MBD2, MECP2) at promoters of CMV, *p16*, *RARB2*, *ESR1*, and *MLH1*. Statistical significance of Bonferroni-corrected *t* tests is shown by *asterisks* (*P < 0.05, **P < 0.01, ***P < 0.001, ****P < 0.0001)

As shown by the gene expression studies above and earlier studies in this model [18, 19], gene transcription cannot be explained solely by DNA hypomethylation, but is more closely related to chromatin remodeling. We therefore examined whether platinum compounds affect histone modifications and methyl-binding occupancy. By Western blots, we found no consistent effects of carboplatin or the combination on global levels of modified histones or expression of methyl-CpG-binding domain protein 2 (MBD2) or methyl-CpG-binding protein 2 (MECP2) (data not shown). We then used chromatin immunoprecipitation (ChIP) and qPCR at five silenced gene loci (*CMV*, *p16*, *ESR1*, *RARß-2*, and *MLH1*) to study this further. For the active chromatin mark H3K4me3 (Fig. 6a), decitabine alone increased enrichment an average of 3.1-fold (range 1.7–4.4), carboplatin increased enrichment an average of 2.3-fold (range 1.7–2.9), while the combination increased enrichment an average of 4.4-fold (range 2.6–5.5-fold, *P* < 0.0001 for the drug effect analyzed by two-way repeated measure ANOVA). Similar results were seen for H3K9Ac, another mark of gene activation. Compared to controls, mean enrichment was 1.7-fold with decitabine, 1.9-fold with carboplatin, and 3.2-fold with the combination (*P* < 0.0001 for the drug effect, Fig. 6b). For silencing histone marks, we found no substantial enrichment for H3K27me3 at these loci, consistent with published data on an inverse correlation between DNA methylation and H3K27me3 in cancer cells [23], and H3K9me2 gave inconsistent results, likely due to the lack of a good antibody (data not shown). We therefore focused on other components of the silencing complex. DNA methylation recruits methyl-binding proteins that are associated with histone deacetylases and a repressive complex, leading to gene silencing. ChIP for MBD2 binding (Fig. 6c) showed reduced enrichment (compared to control) of 0.58-fold, 0.43-fold, and 0.31-fold for decitabine, carboplatin, and the combination, respectively (*P* < 0.0001 for the drug effect, Fig. 6c). For MECP2 (Fig. 6d), we also saw reduced enrichment (compared to control) of 0.77-fold, 0.46-fold, and 0.46-fold for decitabine, carboplatin, and the combination, respectively (*P* < 0.0001 for the drug effect).

### HP1α targeting by carboplatin

**Fig. 7:**
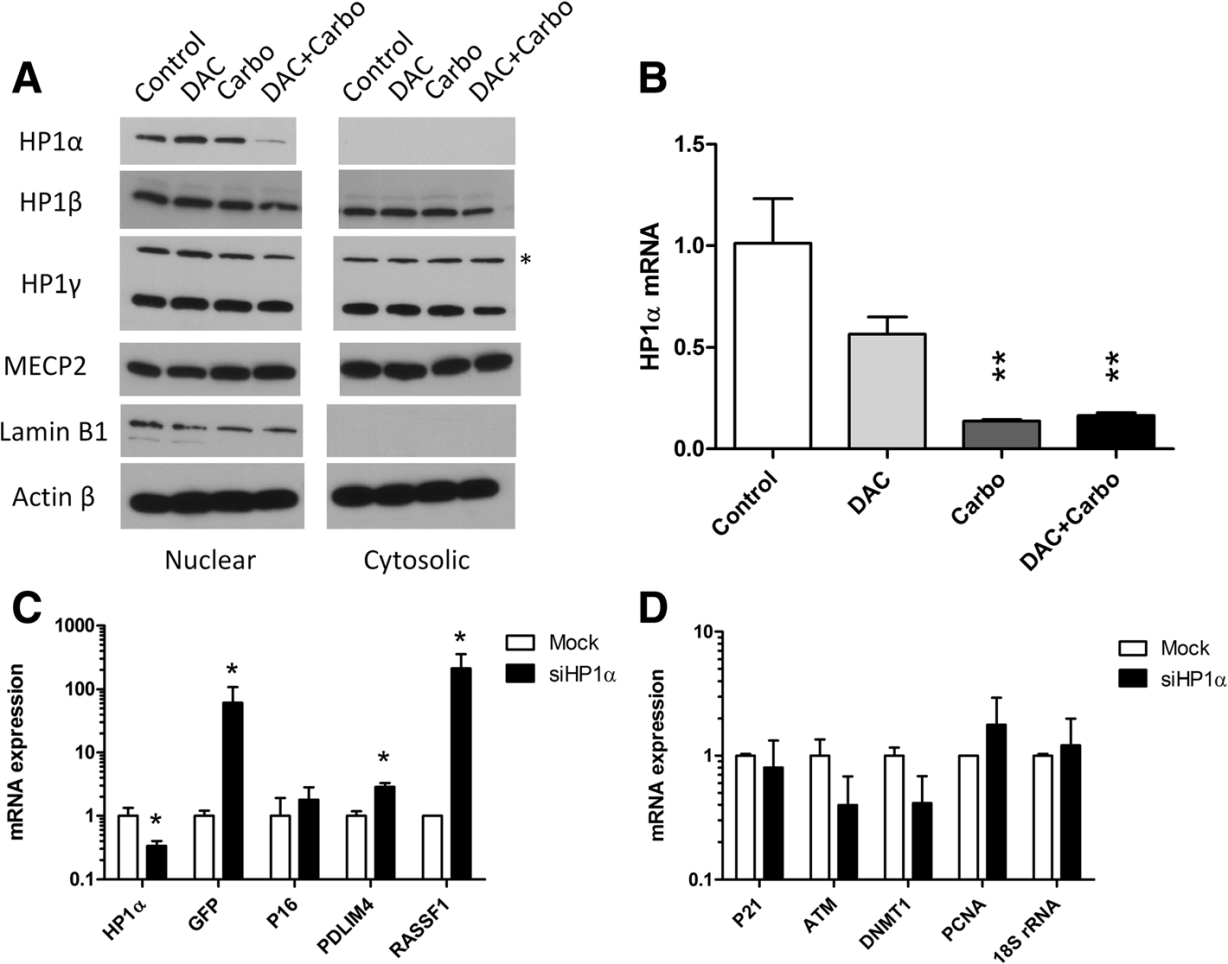
Carboplatin inhibits HP1α in the YB5 Cells. **a**. Effects of drug treatment on the expression of HP1 proteins. We isolated cytosolic and nuclear proteins and performed Western blot analysis of HP1α, HP1β, and HP1γ (* marks a non-specific band). Lamin B1 was used as the internal control for nuclear protein expression. β-actin was used as a control for both nuclear and cytosolic proteins. **b**. Expression of HP1α (*CDX5*) mRNA after drug treatment measured by real-time PCR and normalized to GAPDH. Statistical significance of gene expression changes was analyzed by one-way ANOVA (*P* = 0.005), and Tukey’s multiple comparison tests were used to analyzed the effects of the drug treatments compared to control (**P < 0.01). **c**. siRNA knockdown of HP1α increased expression of genes hypermethylated in the YB5 cells. The expression levels from four experiments were normalized to GAPDH and compared using *t* test (*P < 0.05). **d**. Knockdown of HP1α had little effect on genes with unmethylated promoters (*P* > 0.05)

HP1 is a key mediator of gene silencing induced by promoter DNA methylation. Previous studies indicated shifts in localization of HP1 in response to DNA damage [24]. We therefore examined HP1α, HP1β, and HP1γ expression in nuclear and cytosolic proteins from the YB5 cell line after drug exposure. HP1β and HP1γ were expressed in both nuclear and cytoplasmic fractions and were unaffected by decitabine and/or carboplatin treatment. By contrast, HP1α was only present in the nuclear fraction (Fig. 7a). Treatment with carboplatin alone reduced HP1α protein expression, and the combination of carboplatin and decitabine resulted in marked reduction in HP1α levels in the nucleus (Fig. 7a). This HP1α (*CBX5*) gene repression by carboplatin was also detectable at the level of mRNA expression (Fig. 7b). The data suggested that HP1α repression could be a key mediator of the observed epigenetic synergy. To test this directly, we performed stable short hairpin inhibitory RNA (shRNA) knockdown of HP1α in YB5 cells. Compared with the scrambled shRNA control, knockdown of HP1α partially phenocopied the effects of carboplatin by increasing expression of several hypermethylated genes such as *GFP*, *PDLIM4*, and *RASSF1A* after low-dose decitabine treatment (Fig. 7c). Knockdown of HP1α had little effect on genes with unmethylated promoters (Fig. 7d).

## Discussion

Better therapeutic strategies or targets for drug development are needed to improve the efficiency of epigenetic therapy, expand it to less responsive cancers, and overcome resistance. Here, we used a live cell-based assay to evaluate the epigenetic effects of the hypomethylating drug decitabine in combination with a number of anti-cancer drugs that are currently active in the clinic.

Most of the chemotherapeutics we tested had limited effects when used on their own. In order to increase the effects of low-dose decitabine treatment, we searched for a synergistic combination that would be worth pursuing clinically. The combination of decitabine with the DNA cross-linking agent carboplatin was most effective at gene reactivation of both GFP and endogenous genes. Mechanism wise, we show that both decitabine and carboplatin reduced binding of MBPs to hypermethylated genes as well as inhibited HP1α, resulting in an increase in H3K4me3 and H3K9 acetylation, the active histone marks associated with gene reactivation. The fact that decitabine and platinum analogs exerted their effects through different mechanisms makes this combination intriguing and suggests potential applicability in the clinic. In terms of epigenetic therapy, the most effective drug combination previously reported is DNA methyltransferases inhibitors and HDAC inhibitors [27]. However, the clinical results on this combination have been disappointing in randomized clinical trials to date [28, 8], perhaps because of limited single-agent activity of HDAC inhibitors. Combination therapy using a hypomethylating agent with cytotoxic platinum analogs might be a more effective way to achieve a better response, particularly in solid tumors. Synergy of decitabine with platinum analogs has previously been reported in multiple tumor models [29–31], and the combination of decitabine with carboplatin showed promising effects in several clinical trials in platinum-resistant ovarian cancer [32–35]. This was explained by reactivation of genes silenced by DNA methylation such as the mismatch repair gene *MLH1* [29, 20, 12]. In this study, we suggest that disruption of HP1α binding is an additional mechanism to explain the synergy seen in the combination of decitabine with carboplatin.

It is important to note that we investigated low doses of decitabine in the synergy experiments described here. In contrast to the maximum tolerated dose approach used for conventional cytotoxic therapy in cancer, epigenetic therapy is most effective using low-dose regimens for DNA demethylating agents [25, 26]. Indeed, decitabine used at the low concentration of 25 nM induced significant hypomethylation and gene reactivation in YB5 cells with negligible toxicity and no effect on the cell cycle. This dose is even lower than the peak concentration of decitabine achieved with current treatment regimens, and the data are consistent with our observation in clinical trials that treatment of cancer cells with clinically relevant low doses of decitabine and azacitidine can exert sustained antitumor effects and are less toxic [25, 8]. Nevertheless, we also found that the gene expression response to decitabine at different concentrations was quite variable for different genes, even though the demethylation pattern was very similar. At higher doses, decitabine significantly increased gene expression of some hypermethylated genes without additional effects on DNA methylation. It is possible that decitabine-induced cytotoxicity and DNA damage at high doses might recruit a complex of transcription factors or other pathways, resulting in increased gene reactivation independent of DNA demethylation. These data illustrate the complexity of the dose-response with hypomethylating drugs and point to the potential use of different doses depending on the desired molecular outcome.

We screened the combination of decitabine with several categories of DNA-damaging agents; however, only platinum analogs showed epigenetic synergy. We examined several possible mechanisms for carboplatin-induced gene reactivation. Carboplatin forms interstrand and intrastrand cross-linking with guanine (G), which is likely to influence DNA-binding protein complexes either through blocking DNA-binding sites or changing DNA configuration. However, it is unlikely that this acts on every gene reactivated in enough cells to have a measurable effect on gene expression, leading us to consider indirect mechanisms. We found no direct effects of carboplatin on DNA methylation or bulk histone modifications, but we did document substantial nuclear HP1α depletion, particularly in the decitabine/carboplatin combination. Interestingly, siRNA to HP1α partially phenocopied the effects of carboplatin.

HP1 is a major element in the DNA damage response recruited to the sites of DNA damage [36–38]. Double-strand breaks in DNA promote transient formation of repressive chromatin through loading HP1 [39]. However, our data suggest that, at least for platinum drugs, the opposite effect on HP1α can also be seen. Intriguingly, loosening of HP1 from DNA shortly after induction of DNA damage has been reported [40]. It is possible that with longer time, HP1α is actually degraded, resulting in the depletion we observed. HP1α is part of a central pathway for epigenetic silencing whereby DNA methylation recruits MBPs which in turn recruit HDACs and HMTs. This results in histone H3K9 methylation which triggers HP1 binding, chromatin condensation, and a silenced state that, itself, may trigger more DNA methylation [41-43]. The HP1 variants in mammalian cells include HP1α, HP1β, and HP1γ. However, we observed that carboplatin mainly inhibited HP1α, which was the only purely nuclear HP1 family member. All HP1 proteins share the same architecture that consists of a chromodomain and a chromoshadow domain, connected by a hinge. HP1α shows unique DNA-binding properties in that it binds preformed nuclear chromatin through strong HP1-hinge–DNA interactions rather than through its chromodomain [44]. The DNA-binding activity of the hinge contributes to the high affinity and selective binding of HP1α to tetranucleosomes containing H3K9me3 [45]. This unique binding pattern may explain the selective degradation of HP1α by DNA cross-linking. It is also worth noting that the non-cross-linking O6-guanine methylating agent temozolomide [46] also showed synergistic reactivation of GFP expression in YB5 cells at high doses (Table 1). Carboplatin has been shown to deplete methylguanine methyltransferase [47], and it is therefore possible that an increase in genome-wide O6-guanine methylation may be involved in the observed gene reactivation.

## Conclusions

In summary, using this live cell screening system, we uncovered unexpected epigenetic synergy between decitabine and platinum analogs which is worth pursuing clinically. While decitabine is currently being used as a “priming” therapy to sensitize to platinum, our data suggest that the two drugs could potentially be administered concurrently to maximize dual cytotoxic and epigenetic effects.

## Availability of supporting data

The data discussed in this publication have been deposited in NCBI’s Gene Expression Omnibus [58] and are accessible through GEO Series accession number GSE66296.

## Methods

### Cell culture and treatment protocols

The human colon cancer cell line SW48 and leukemia cell lines HL60 and K562 were obtained from American Type of Culture Collection. SW48 was grown in L-15 medium plus 10 % fetal calf serum (FCS) in plastic tissue culture plates in a humidified atmosphere containing 1 % CO_2_ at 37 °C. Leukemia cell lines were grown in RPMI 1640 medium supplemented with 10 % FCS in a humidified atmosphere containing 5 % CO_2_ at 37 °C. For the growth inhibition assay, cells were split at a density of 1 × 10^5^/mL in 5 mL of medium 24 h before treatment. Different concentrations of drugs were added to the medium either separately or in combination. The doses that inhibited 50 % proliferation (IC50) were analyzed by the median-effect method. We used fixed ratios of 1/8, 2/8, 3/8, 4/8, 6/8, and 8/8 IC50 for decitabine and carboplatin for each respective cell line. In vitro cytotoxicity was assayed in triplicate by the following experimental conditions: control, decitabine, tested drug (listed in Table 1), and decitabine + tested drug. The proportion of live cells in treated plates was measured by counting cells in hemocytometer based on trypan blue exclusion. The effects of combinations were estimated using the CalcuSyn software (Biosoft) based on the median-effect method by Chou and Talalay [48].

### FACS analysis

GFP expressing cell percentages were measured using a Becton Dickinson FACSCalibur Flow Cytometer at the University of Texas MD Anderson Cancer Center Flow Cytometry Core Facility. For GFP reactivation after a 96-h incubation with daily decitabine, the cells were trypsinized and resuspended in growth medium. Cell cycle analysis was performed by DNA quantitation after propidium iodide staining. Flow cytometry data were processed using FlowJo software (Tree Star, Inc, Ashland, OR).

### Bisulfite pyrosequencing for methylation analysis

Bisulfite treatment was performed as reported previously [49, 50]. Genomic DNA was denatured by 0.2 M NaOH at 37 °C for 10 min followed by incubation with freshly prepared 30 μL of 10 mM hydroquinone and 520 μL of 3 M sodium bisulfite (pH 5.0) at 50 °C for 16 h. DNA was purified with a Wizard Miniprep Column (Promega, Madison, WI), desulfonated with 0.3 M NaOH at 25 °C for 5 min, precipitated with 2.5 M ammonium acetate and ethanol, and dissolved in 50 μL of TE buffer (Tris-HCl 10 mM, EDTA 1 mM, pH 8.0). Bisulfite-treated DNA (40–80 ng) was amplified with gene-specific primers by polymerase chain reaction (PCR). Primer sequences for the five genes and LINE-1 elements analyzed are shown in Additional file 2: Table S4. We measured levels of DNA methylation as the percentage of bisulfite-resistant cytosines at CpG sites by pyrosequencing with the PSQ HS 96 Pyrosequencing System (Biotage, Charlottesville, VA) and Pyro Gold CDT Reagents (Biotage) as previously described [51].

### RNA extraction and cDNA synthesis

Total cellular RNA was extracted by TRIzol reagent (Life Technologies). RNA was eluted with RNase-free water, quantified by spectrophotometry and used for first-strand complementary DNA (cDNA) synthesis according to the manufacturer’s protocol (Applied Biosystems). Three micrograms of RNA was reversely transcribed to single-stranded cDNA. The reverse transcription was performed in a total volume of 50 μL containing 0.2 mM of each dNTP (Amersham Pharmacia Biotech, Piscataway, NJ), 10 μM of random hexanucleotide primers (Invitrogen), 200 U Moloney murine leukemia virus reverse transcriptase (M-MLV RT) (Promega, Madison, WI), and 25 U RNAsin (Promega) at 37 °C for 2 h. The obtained cDNA was stored at −80 °C.

### Quantitative reverse transcription-PCR

Real-time quantitative reverse transcription-PCR (qRT-PCR) was done with the ABI 7000 Sequence Detector (Applied Biosystems). We used ready-made TaqMan® assays on the analyzed genes and custom-designed GAPDH primers and TaqMan® probe [52] (Applied Biosystems). Reactions for qRT-PCR were done with the TaqMan® universal PCR Master Mix kit (Applied Biosystems) in 96-well plates. Each sample was measured in triplicate. PCR was run using the following conditions: an initial denaturation step of 95 °C for 10 min followed by 40 cycles at 95 °C for 15 s and 60 °C for 1 min. The resulting data were analyzed with ABI Prism 7000 SDS software (Applied Biosystems). The threshold cycles (CT) were determined, and the differences in the CT values for GAPDH and selected genes were calculated.

### Chromatin immunoprecipitation

Chromatin immunoprecipitation (ChIP) analyses were performed as described previously [31]. Briefly, cells were fixed in 1 % formaldehyde and lysed followed by sonication shearing using the Bioruptor sonicator (Diagenode, Belgium). After centrifugation, the soluble chromatin was subjected to immunoprecipitation with antibodies against different modified histones. Antibodies used were directed against histone H3 (ab1791, Abcam, Cambridge, MA), histone H3K9 acetylation (07-030, Millipore, Billerica, MA), trimethyl-histone H3 (Lys4) antibody (04-745, Millipore), MeCP2 (ABE171, Millipore), MBD2 (ab38646, Abcam), histone H3K27 trimethylation (07-449, Millipore), and IgG (sc-2027, Santa Cruz Biotechnology, Santa Cruz, CA) as a negative control. The complexes were drawn off with protein A-agarose and G-agarose beads (ratio 3:1) and washed sequentially with low-salt, high-salt, LiCl, and Tris-EDTA buffers and were finally extracted with freshly prepared 1 % SDS-0.1 M NaHCO_3_. Samples were brought to 65 °C for 4 h to reverse DNA and protein cross-links, and DNA was then purified with a Qiagen DNA extraction kit. 1.0 × 10^6^ cells were used per antibody pulldown. Quantitative analyses were done by qPCR using 6-FAM-labeled probes targeting three regions of the CMV-EGFP locus (Applied Biosystems). Sequences of all primers and probes are in Additional file 2: Table S5.

### Cell lysate and histone preparation and Western blots

Total histones were prepared by acidic extraction and resolved on 15 % SDS-polyacrylamide gels as described [44]. Cell lysates of nuclear and cytosolic proteins were prepared using NE-PER™ kit (78833, Pierce). Antibodies used for Western blotting were directed against pan-acetylated histone H4 (06-866, Millipore), histone H4 (07-108, Millipore), histone H3 (ab1791, Abcam), trimethyl-histone H3 (Lys4) antibody (04-745, Millipore), histone H3K9 acetylation (07-030, Millipore), histone H3K27 trimethylation (07-449, Millipore), HP1α (ab77256, Abcam), HP1β (ab10478, Abcam), HP1γ (ab10480, Abcam), MeCP2 (ABE171, Millipore), MBD2 (ab38646, Abcam), and lamin B1 (ab16048, Abcam).

### Gene expression microarrays

Gene expression microarray analyses were conducted using the Agilent Whole Genome 4x44K v2 Microarray (G4112F). Hybridized arrays were scanned using the Agilent G2505B Scanner and processed in R using Bioconductor packages geneplotter, limma, and agilp [53, 54].

### Digital restriction enzyme analysis of methylation

Genome-wide analysis of DNA methylation by digital restriction enzyme analysis of methylation (DREAM) was done as previously described [21]. Briefly, 5 μg of SW48 genomic DNA spiked in with 10 pg of methylation standards were digested with 100 units of *Sma*I endonuclease (NEB) for 3 h at 25 °C. Subsequently, 100 units of *Xma*I endonuclease (NEB) were added and the digestion was continued for an additional 16 h at 37 °C. Digested DNA was purified and incubated for 30 min at 37 °C with 15 units of Klenow Fragment 3′ → 5′ exo-DNA polymerase (NEB) to fill in the recesses and add dA overhangs at 3′ ends of the DNA fragments. Illumina paired-end sequencing adapters were then ligated using Rapid T4 DNA ligase (Enzymatics). The ligation mix was size selected by electrophoresis in 2 % agarose, and fragments with apparent sizes of 250–350 and 350–500 bp were separately amplified with Illumina paired-end PCR primers, iProof high-fidelity DNA polymerase (Bio-Rad Laboratories) master mix, and 18 cycles of amplification. The resulting libraries were purified with AMPure magnetic beads (Agencourt). The libraries were sequenced by paired-end 36 nt sequencing on Illumina HiSeq 2000 at the MD Anderson Center for Cancer Epigenetics. After mapping the sequencing reads to the reference human genome (hg18), we determined methylation levels at target CpG sites as described previously [21]. The results were assembled for further analysis in the Microsoft Access relational database containing the full annotation of all *Sma*I/*Xma*I sites in the human genome.

### HP1α knockdown

siRNA oligo GGAUUGCCCUGAGCUAAUUUU (Ambion) was transfected to YB5 cells using Lipofectamine RNAiMAX reagent (Life Technologies) following the manufacturer’s protocol. Mock transfection with Lipofectamine only was used as the control.

### Statistics

GraphPad Prism 5 software was used for statistical analyses. Two-way ANOVA was used to test for effects of drug treatment, and *t* tests with Bonferroni correction for multiple comparisons were used to assess the effects on individual genes. One-way ANOVA with Tukey’s multiple comparison post-tests was used for the analysis of gene expression changes. The Spearman method was used to assess correlation between DNA methylation and gene expression. Two-tailed *P* < 0.05 was considered a significant difference. Chi-square test was used for the numbers of regulated genes. Functional gene annotation, enrichment analysis was performed using GeneCodis online tool (http://genecodis.cnb.csic.es) [55–57].

## Additional files

Additional file 1: Table S1. Top functional annotation categories of genes upregulated by carboplatin. **Table S2.** Top functional annotation categories of genes downregulated by carboplatin. **Table S3.** Functional annotation of genes upregulated by decitabine and carboplatin (DAC+Carbo). Additional file 2: Table S4. Primers for bisulfite pyrosequencing.**Table S5.** Primers for ChIP Q-PCR.

## Abbreviations

Carbo: carboplatin
ChIP: chromatin immunoprecipitation
CI: combination index
CMV: cytomegalovirus
CT: PCR cycle at threshold fluorescence
DAC: 5-aza-2′-deoxycytidine
DREAM: digital restriction enzyme analysis of methylation
FCS: fetal calf serum
H3K4: histone H3 lysine 4
H3K9: histone H3 lysine 9
HDAC: histone deacetylase
HP1: heterochromatin protein 1
IC50: inhibitory concentration giving 50 % inhibition
LINE-1: long interspersed nuclear element 1
MBD2: methyl-CpG-binding domain protein 2
MBP: methyl-binding protein
MDS: myelodysplastic syndrome
MECP2: methyl-CpG-binding protein 2 (Rett syndrome)
qRT-PCR: quantitative reverse transcription polymerase chain reaction
shRNA: short hairpin inhibitory RNA
