# Supplementary material for: Epigenetic synergy between decitabine and platinum derivatives
Source: Clin Epigenetics. 2015 Sep 11;7(1):97. doi: 10.1186/s13148-015-0131-z (PMC4567801; doi:10.1186/s13148-015-0131-z)
Supplement: Additional file 1: Table S1. — Top functional annotation categories of genes upregulated by carboplatin. Table S2. Top functional annotation categories of genes downregulated by carboplatin. Table S3. Functional annotation of genes upregulated by decitabine and carboplatin (DAC+Carbo). [file 13148_2015_131_MOESM1_ESM.pdf]

Table S1. Top functional annotation categories of genes upregulated by carboplatin.

Functional category	Number of genes	P value*	Genes
mitochondrion	111	0.002	ALDH1B1, CAV1, L2HGDH, GRPEL1, C8orf38, C9orf46, PRODH, TAZ, ATP5O, VDAC2, DCAKD, MTCP1NB, MRPS6, NDUFC1, MRPS35, MRPL40, GLOD4, MPV17, GPX1, PAX9, BCL2L1, IARS2, ACAT1, TYMS, CRLS1, PTPMT1, FKBP8, MECR, DDIT4, AATK, RFK, AGXT2L2, SLC25A37, BAK1, SFXN4, NDUFAF3, GOT2, UNG, C1orf151, FAM82A2, IDH1, PMAIP1, RHOT2, MRPS36, ABCD3, SLC25A20, METTL12, DLD, MRPS15, MRPL10, PISD, PROSC, MCL1, ACOT8, AUH, CLTC, COX8A, IREB2, SLC25A46, ATP5L, AIFM2, PRKACA, C17orf89, SNN, BBC3, SQRDL, RILP, BNIP3L, MRPL38, IDI1, ECSIT, SPATA18, UXS1, SDSL, POLRMT, GTPBP3, NUDT8, NDUFA8, XRCC3, ETHE1, DNLZ, BRI3BP, GLS2, RAB35, MTG1, SLC25A17, ABCB6, BRP44L, JTB, NLRX1, ECH1, GRAMD4, YRDC, ME1, HAX1, FTSJ2, SCO1, TIMM13, VPS25, NOP14, AFG3L2, NDUFA3, PSMA6, SLC22A4, MYCBP, MRP63, ATP5I, ZBTB9, FXN, FDXR, KIAA0141
apoptotic process	48	0.033	CYFIP2, PHLDA1, IER3, ZMAT3, PSMB4, SGMS1, CCAR1, CLSPN, PPP1R15A, HTT, PLK3, BCL2L1, ARHGEF7, MFSD10, FKBP8, RNF41, DDIT4, AATK, MDM4, BAK1, PHLDA3, DRAM1, EGLN3, SGPP1, MKNK2, FAM82A2, PMAIP1, RHOT2, MCL1, AEN, NTN1, BBC3, ECE1, LGALS7B, GADD45A, EPHA2, ITM2B, AXIN1, TNFRSF12A, TNFRSF10B, GRAMD4, PSMD11, PSMB10, TGFBR2, PSMA6, TNFRSF10D, PAK6, KIAA0141

*hypergeometric P value corrected for multiple testing

Table S2. Top functional annotation categories of genes downregulated by carboplatin.

Functional category	Number of genes	P value*	Genes
cell division	25	0.00004	CENPF, ANXA11, FBXO5, PDS5B, PTTG1, OIP5, NCAPH, LMLN, PHF13, HAUS5, POGZ, UBE2C, NCAPD2, KIAA1009, ASPM, CDC20, BUB3, CCNB2, BUB1, CIT, KIF20B, CEP55, CKS2, SPAG5, CCNA2
cell cycle	32	0.00008	UHRF1, CDKN1B, ANXA11, CHAF1A, RAB11B, RPS6KA3, GAS1, PDS5B, OIP5, CDKN3, MAP3K8, NCAPH, LMLN, HAUS5, LIN9, POGZ, GSG2, PRC1, UBE2C, NCAPD2, KIAA1009, MELK, E2F8, ARL3, ASPM, CDC20, CIT, KIF20B, CEP55, CKS2, C15orf42, SPAG5
DNA replication	17	0.00036	CENPF, POLQ, CHAF1A, POLD3, RFC3, POLA1, POLE2, TK1, BLM, MCM9, LIN9, GINS2, DUT, RMI1, TOP2A, GINS3, C15orf42

*hypergeometric P value corrected for multiple testing

Table S3. Functional annotation of genes upregulated by decitabine and carboplatin (DAC+Carbo).

Functional category	Number of genes	P value*	Genes
cell adhesion	6	0.00423	HES5, IGFBP7, PSTPIP1, MYBPC3, THBS1, COL5A1
apoptotic process	5	0.04282	TNFRSF10C, PLEKHF1, PREX1, PDIA2, NGFRAP1
integrin signalling	3	0.00015	COL9A2, COL5A1, COL8A2

*hypergeometric P value corrected for multiple testing
