# Supplementary material for: Epigenetic synergy between decitabine and platinum derivatives
Source: Clin Epigenetics. 2015 Sep 11;7(1):97. doi: 10.1186/s13148-015-0131-z (PMC4567801; doi:10.1186/s13148-015-0131-z)
Supplement: Additional file 2: Table S4. — Primers for bisulfite pyrosequencing.Table S5. Primers for ChIP Q-PCR. [file 13148_2015_131_MOESM2_ESM.pdf]

Table S4. Primers for bisulfite pyrosequencing.

Name of Gene	Primer Type*	Sequence
CMV	F	GGGGTGGAGATTTGGAAATT
	RU	*GGGACACCGCTGATCGTTTA*CCCACTTAACAATACATCAAATATATCA
	S	TGGAGATTTGGAAATTTT
P16	F	GGTTGTTTTYGGTTGGTGTTTT
	RU	*GGGACACCGCTGATCGTTTA*ACCCTATCCCTCAAATCCT
	S	AGTTAGGTGTGGGATATAGT
PGR	F	TGTGGGTGGTATTTTTAATGAGA
	RU	*GGGACACCGCTGATCGTTTA*CCCCCTCACTAAAACCCTAAA
	S	GGGATTTGAGATTTT
MLH1	F1	TTGAGTTAGATTATTTTAGTAGAGGTATAT
	F2	GGTATTTTTGTTTTTATTGGTTGGATATTT
	RU	*GGGACACCGCTGATCGTTTA*CCAATCAAATTTCTCAACTCTATAAATTACTA
	S	TTTAAAAAAGAATTAATAGGAA
PDLIM4	F	TTTGTGAGTTTGGATTGGT
	RU	*GGGACACCGCTGATCGTTTA*CCCCAAATAAACCCTCCAT
	S	TTTATTTAGTTTTTAGAGAT
NPM2	F	TTTTTAGGTTAGAGGGGATGAG
	RU	*GGGACACCGCTGATCGTTTA*TAAACCTAACTCAAAAACCTCTATA
	S	GTTAGAGGGGATGAGGT
OLIG2	F	TTTTAAAGGTGAGGATGTTTATTAT
	RU	*GGGACACCGCTGATCGTTTA*AAAAATCCAAACCCCCTATAT
	S	AGGTGAGGATGTTTATTATA
LINE-1	F	TTTTGAGTTAGGTGTGGGATATA
	Bio-R	Biotin-AAAATCAAAAAATTCCCTTTC
Biotin-Universal	BU	Biotin-*GGGACACCGCTGATCGTTTA*

Primer type: F, forward; RU, reverse primer with the 5'-*GGGACACCGCTGATCGTTTA* universal tag; BU, universal primer with 5'-biotin for biotin labeling of the reverse strands; S, pyrosequencing primer.

Table S5. Primers for ChIP Q-PCR.

Name of Gene	Primer Type	Sequence 5'→ 3'
GFP	Forward<br>Reverse<br>Probe*	CATCTACGTATTAGTCATCGCTATTACCA<br>TCAATGGGCGTGGATAG<br>6-FAM GAAATCCCCGTGAGTCAAACC
MLH1	Forward<br>Reverse<br>Probe*	TTCCGTTGAGCATCTAGACGTTT<br>CCTGCCACGAACGACATTTT<br>6-FAMCTTGGCTCTTCTGGCGC
P16	Forward<br>Reverse<br>Probe*	CCCGCCCCGTTTCCT<br>CCGATTCAATTTGGCAGTTAGG<br>6-FAMTCCGCGATACAACCT
RARβ-2	Forward<br>Reverse<br>Probe*	TTGGAAGGAGAACTTGGGATCTT<br>AGGCTTGCTCGGCCAATC<br>6-FAMCTGGGAACCCCCC
ESR1	Forward<br>Reverse<br>Probe*	GCGGCCACGGACCAT<br>TAGGGCCATCCCAGATGCT<br>6-FAMCCATGACCCTCCACACC

*The TaqMan® probes were labeled with 6-FAM™ on the 5' end and with minor groove binder (MGB) and nonfluorescent quencher (NFQ) on the 3' end.
